# From Our Correspondents

**Published:** 1983-07

**Authors:** 


### From Our Correspondents

**Obstetrics in General Practice**

The past two decades have seen a marked reduction in obstetric care by general practitioners. Most general practitioners now confine their care to antenatal and postnatal consultations, avoiding any involvement in the delivery.

This change has been hastened by the use of deputising services, closure of general practitioner units, the increasing size of hospital teams together with what can be termed 'active obstetric management'. No longer do mothers labour for more than 24 hours; epidurals and continuous monitoring of foetal well-being are common. Obstetrics has become the province of the young doctor; in major units it is nowadays uncommon to see a doctor over 35 years of age being involved in the management of labour – be he consultant obstetrician or general practitioner.

General practitioners have to ask themselves what is to be gained by being involved in intrapartum care. It is certainly not financially worthwhile – the confinement fee being about £13 – and it can certainly be disruptive to normal surgeries as not all deliveries occur at night! The positive gains from general practitioner involvement include a deepening doctor–patient relationship and the satisfaction of being involved in one of nature's miracles.

In Bristol general practitioner deliveries form a small part of the total. In the Bristol Maternity Hospital there are never more than 100 GP deliveries a year (rather more at Southmead).

A questionnaire was circulated to all general practitioners in Avon in 1983 asking for information about their obstetric commitment. About 250 general practitioners (out of nearly 400) replied and 42% of these claim to be providing intrapartum care; most of the remainder being involved in antenatal and postnatal care only. Of those providing intrapartum care 60 practise within Bristol (one-third of whom are willing to be involved in home deliveries).

General practitioner obstetrics seems then to be alive, is certainly geographically patchy and hopefully will continue to provide mothers with an alternative form of delivery to the largely impersonal hospital delivery system.

M.W.

**Meetings of National Societies in Bristol**

This April, Bristol was host to two well-known medical societies – the British Cardiac Society and the Association of Physicians of Great Britain and Ireland. The former began as the Cardiac Club in 1922 and started the habit of meeting the day before the Association of Physicians. Both societies move around the university centres in the United Kingdom and last met in Bristol in 1961. This year the meeting was chaired by Dr. Bill Barritt and met in the Union building, the dinner being held in the Grand Hotel. The Association of Physicians (founded in 1907) was chaired by Professor Alan Read in the Physics lecture theatre. There was a mayoral reception in the City museum on the Thursday evening followed by a supper in the Great Hall of the University to the accompaniment of organ music. The annual dinner was held in the Council House, where the chief guest was the President of the Royal College of Surgeons.

Both meetings were a great success, with many papers and demonstrations being provided by the home team. Fine weather made the meetings especially memorable, and many of the ladies enjoyed a trip to Bath and a tour of the centre of Bristol on foot. Our city has a great deal to offer visitors, and is becoming increasingly popular as a conference centre. Sponsorship of such medical meetings is now becoming the norm. Providing the support is 'low key' and does not interfere with scientific independence, I can see no objection, though I admit to being a convert to this view. By defraying the enormous overhead costs of meetings, doctors in training and junior consultants can attend, often with their wives. I believe very strongly that encouraging friendship between doctors is important, and would not like to see a return to the pre-war rivalry and enmity which was so rife between general practitioners in the same area, and between 'honoraries' on the staff of a hospital, or the prejudice of teaching hospital staff against the staff of other hospitals.

Since the war, there has been a great levelling upwards of facilities and quality of staff in hospitals, particularly those not graced by the prefix 'teaching'. We have two examples of such high quality consultants retiring this summer. Bob Craig, after training at Guy's and the Brompton, has given 35 years of devoted service to the chest service in Bristol, working mainly at the central clinic, Ham Green, Southmead and Weston. Francis Page, who has trained at the Middlesex and Central Middlesex, has given stalwart service to medicine and neurology mainly at Southmead and Cossham. He has also been an inspired teacher of both students and postgraduates, even though working in a nominally non-teaching hospital! Both will be missed by their patients and colleagues.

H.G.M.

**British Journal of Surgery**

After a fruitful association for the past 70 years the *British Journal of Surgery* and John Wright & Sons are going their separate ways. Since Ernest Hey

Groves was the first Editor of this *Journal*, it is scarcely surprising that a Bristol firm was chosen to publish and print his brainchild. The *Journal* has been jointly owned by Wrights and the British Journal of Surgery Society, an arrangement that can produce conflicting interests in the modern commercial world. The Society has therefore acquired total ownership of the *Journal*, in trust for British Surgery; from 1984 the monthly issues will be published elsewhere. As somebody caught in the crossfire during this separation, your correspondent welcomes the recent good sense of our own centenarian, the *Bristol Medico-Chirurgical Journal*, in deciding to be printed by John Wright & Sons.

The Bristol Royal Infirmary is featured on the cover of the March 1983 issue of the *British Journal of Surgery*. Mr. Albany Wiseman's drawing shows that hazardous nexus of the hospital, the Marlborough Street pedestrian crossing, on a pleasant backing of powder blue; it is accompanied by an irreverent thumbnail history of the Infirmary. If earlier examples from this series of eminent British hospitals do well, we hope that coloured lithographs of the Infirmary may become available for purchase in due course.

R.C.N.W.

**Frenchay Hospital**

On Sunday, 12th June, on what appeared to be the first day of glorious summer sunshine, a superbly organised and picturesque Strawberry Fair and Marathon Drive took place in, and from, the grounds of Frenchay Hospital. This event was organised by the League of Friends of Frenchay Hospital, and it was wonderful news to hear later on that about £1250 was raised for the benefit of the hospital, a sum which will be used to purchase specialised equipment.

As a result of witnessing such a well-organised and delightful display of horses, ponies, gigs and traps, as well as the Fair, it was a pleasure for your correspondent to enquire further into the origins and activities of the League, as we medical members of the hospital gain enormously from their hard work and generosity, and do not always remember how their funds arise.

Information was therefore sought from one of their Joint Hon. Secretaries who attends the hospital regularly about once a week. From a diary which is issued in the name of the Friends of Frenchay Hospital, the following information is obtained from an introductory preface written by their Chairman, Eric Godfrey.

'THE FRIENDS OF FRENCHAY HOSPITAL are a purely voluntary body, formed in 1960, to help provide better facilities for Patients, Staff and Visitors in Frenchay Hospital. The Friends have raised over £70,000 since their formation and have used the money to provide a Playroom on the Children's Burns Unit, Dayrooms on Wards 7 and 8, furnishings in the Dayroom on Ward 15 and in the Nurses Rest Room, a Gamma Counter for the Pathology Laboratory and a Mother and Baby Unit on Ward 8. In addition to providing the money for these major projects, the Friends have donated equipment for several wards, hardship grants for Patients' relatives, Christmas presents to every patient in Frenchay Hospital every Christmas, and help in many other directions.

'I think it is true to say that the one incident which brought the greatest satisfaction to the Friends was the 22 hours long operation to sew back the severed fingers of an injured factory worker. This very skilled operation would not have been possible without using the operating microscope provided by the Friends at considerable cost. Another highlight was the gift of a Curapuls machine (£2,600) to the Physiotherapy Department.

'To celebrate the Golden Jubilee of the Hospital in 1981 the Friends gave a blood gas Machine to the Respiratory Unit for the study of chronic chest disease at a cost of £13,500.

'Money is raised by donations from expatients, in lieu of flowers, gymkhanas, sponsored swims, whist drives, wine and cheese parties, jumble sales, Christmas Fairs, etc., etc.'

In relation to the Horse and Pony Show side of the Sunday Fair, it is of interest to recall that a Pony Show and Gymkhana have been organised each year for the past 25 years, and for several years a Marathon Drive has been incorporated with a great deal of help from Mr. and Mrs. S. I. Roberts and their family. Last year the Gymkhana had to be discontinued, but the Marathon Drive took place and this year a Strawberry Fair was incorporated. Mr. Doggrell officiated as Judge and the Scutt Cup was awarded to Mrs. Pat Baker.

A letter of appreciation was sent to Mrs. Phyllis Candler, Executive Secretary of the British Driving Society, in acknowledging the help given by their members. This is part of the British Horse Society which was founded in 1957 to encourage and assist those interested in driving horses and ponies. The Patron is the Duke of Edinburgh.

A further quote about this Society is of interest:

'Until recently the Headquarters of the Avon Branch of the B.H.S. was at Dodington House, where the Area Commissioner had his office. Meets and drives are arranged in the summer months, and Film Shows and Meetings during the winter.

'There are Driving Classes at all the Shows all over the country during the season. The Annual

British Driving Society Show is taking place this weekend (June 18th) at Smiths Lawns at Windsor.

'The competitors are judged on many things including the Vehicle, Dress, Ability to Drive, Signals, before and after an 8 mile drive. The drive from Frenchay Hospital Grounds went through Hambrook, Moorend, Winterbourne Down, Winterbourne, where a break for refreshments was made, and a return to the Hospital.'

It is hoped that this information about our League of Friends proves of interest to readers of this article. The information has certainly revealed to your contributor what a marvellous group of Friends we have at Frenchay Hospital. I am sure we would like them to know how much we appreciate them.

G.T.

**Southmead Hospital**

Now that Spring is slowly emerging into Summer the gardens in the hospitals of the Southmead District are an everchanging delight both to the patients and the staff. During the last month three 'walkabouts' have taken place around the gardens at Southmead, Brentry and Ham Green Hospitals. These have been under the expert guidance of Tom Havill, the Superintendent of gardens to the Southmead District, and to the surprise of the organisers have been a great success. A happy feature of all these tours was the wide range of people who took part. From the very young (9 weeks old) to the elderly and retired; from nurses and technicians to secretaries, administrators and doctors, all came, learnt and admired. The sole common denominator of the participants was some interest in gardens and some connection with Southmead. In these days of large impersonal organisations, such gatherings from all levels of employment within the NHS are a rare and happy occasion. It should be added that, apart from learning about plants and trees, we learnt from Tom some of the challenges and difficulties of managing the gardens.

What were the most memorable features?

At *Southmead* the primary difficulty in creating the gardens has been the constant alterations required due to both expansion and destruction of buildings. Where John Milton Ward stood for so many years a huge concrete foundation had to be removed before trees could be planted and the lovely new beds created. Some trees, often large, have been successfully moved many times. The individual garden bays between the wards have all been planned with care and often include trees and shrubs donated by old patients. We all discovered small corners we had never visited before, including a lovely sheltered courtyard in 'B' Block where exotic shrubs thrived.

At *Brentry* we were amazed at the size of the gardens, sadly with scarcely a patient to be seen and, due to lack of staff, now largely confined to trees and lawns. The mature trees near the main house were much admired, including a lovely old gnarled mulberry. We learnt that large old trees are a severe financial burden as, since Health and Safety regulations forbid gardeners to climb trees, tree surgeons, at £200 a tree, had to be employed to operate following wind damage to the large cedars. The disappearance of newly planted trees and plants is a problem in all the unguarded gardens and 150 new yews planted by the main road vanished so fast that the survivors had to be removed. The most exciting feature was the greenhouses where production of all the bedding and house plants for the whole District is now centralised. A profusion of geraniums, begonias, fuschias and many other plants were much admired.

At *Ham Green* it was again the mature cedars and other large specimen trees around the old house that made the first impact. Planning has to span both the bedding plants for this year and also includes the siting of new trees which will only be mature in 150 years. The Judas trees, with their pink blossoms sprouting from their stems, were an exciting novelty to all but the most knowledgeable. We dallied happily in Kay Iles' Memorial Garden by the Chapel and appreciated the loving expertise that had gone into its creation. Surely we shall all enjoy seeing it develop and mature over the years. The gardens at Orchard View (the resident centre for the young chronic sick) were admired. Here at last patients were benefiting from working in the gardens – surely one of the best types of occupational therapy. Lastly, and possibly most exciting to the tree lovers amongst us, we came to the southern border of the gardens to find that 400 English hardwoods (at 30p a tree) had been planted last year. These had not been vandalised and were thriving – surely the best long-sighted investment for the future.

I think that all who joined in any of these walkabouts enjoyed themselves more than they could have anticipated and were just a little jealous of Tom and his colleagues who have such a creative job and who can, and do, give much pleasure to so many people. We anticipate that these walkabouts will become a regular feature in the Southmead scene.

C.R.T.

**Ham Green Hospital**
**Recent Retirements**
**Dr. Francis Page**

Francis Page came to Bristol in August 1958 from the Central Middlesex Hospital, and very quickly continued his great interest in teaching both at post-graduate and undergraduate level. His enthusiasm and teaching ability played a large part in Southmead Hospital being recognised by the University for Undergraduate training in Medicine and Surgery and he became the first Clinical Dean at the Hospital in 1968. He played a large part, with other colleagues, in the development of the Postgraduate Centre for Medical Education at Southmead Hospital.

In 1974 Dr. Page accepted an additional challenge with the opening in December of that year of the Young Chronic Sick Unit, Orchard View in the grounds of Ham Green Hospital.

Since then he has been very closely associated with the work of the Unit.

Another example of his life-long interest in helping people is his association, with his wife, in the work of St. Peter's Hospice in caring for the terminally ill.

Dr. Page retired in May. We welcome his successor Dr. Iaian Ferguson to Bristol and to Ham Green especially.

**Dr. Robert Craig**

Bob Craig, who retired in June, came to Bristol via the Brompton Hospital and Guys in June 1950 and very quickly established a reputation in the field of Chest Medicine with tuberculosis playing a large part of his work in the early days especially in the Chest Clinics and Ham Green Hospital where most of the T.B. beds were situated, apart from some at Charterhouse.

His skill in interpreting chest X-rays is well known and I feel he can usually recognise his patients just as easily by their X-ray as he can by their features.

Over the years at Ham Green, with his undoubted interest in fine food and wine, it has become a tradition that towards the end of one's appointment as his houseman, the said houseman is encouraged to produce a cake, which is duly consumed during the course of a ward round. Most have been baked by the housemen themselves, others came by various routes.

During his years as a physician, Dr. Craig has always exhibited considerable skill as an administrator.

At the same time we welcome his successor Dr. John Harvey and his family. John is already well known to us because of his membership of the Area Chest Physicians Group who meet monthly in Ham Green.

**Teaching**

Students, usually in pairs, continue to come to Ham Green for a week at a time as part of their training during their supplementary attachment at Ham Green. They come from both this country and abroad to do their elective period.

During this last session two symposia proved very successful and were very well attended.

The first, on 'Psychiatry in the elderly', was organised by Dr. Ihsanul Mian, Consultant Psychiatrist and colleagues; the other on 'Problems in the Elderly' was organised by Dr. Geoffrey Burston, Consultant Geriatrician. Both attracted speakers and an audience from over a wide area. Further symposia are in the process of being organised for next term. Dr. Stewart Glover has organised a course in infectious diseases as part of the MRCP Course.

D.W.W.

**Stoke Park Hospital**

Fifty years ago, the results and the value of the global approach to the problem of Mental Handicap, as demonstrated by many scientific papers published by the medical and other staff of Stoke Park Hospital, appeared in the first edition of *Stoke Park Studies*, under the editorship of Professor R. J. A. Berry in 1933. Since then, a second edition and a supplement to *Stoke Park Studies* have been published.

To commemorate the pioneers and researchers in this field, four new wards were named after them. Two new geriatric wards were opened last year by Mr. Hugh Rossi, Minister of Social Security and Disabled and these were named after Mrs. Burden, co-founder of Stoke Park Hospital and Professor Berry, first director of medical services and researcher.

Last April, two other wards were opened by the President of the Royal College of Psychiatrists, Professor K. Rawnsley. The intensive care unit was named after Dr. R. M. Norman, medical superintendent and world-renowned neuro-pathologist, and the pre-discharge house was named after Dr. Fraser Roberts, Fellow of the Royal Society and a great authority on medical genetics.

Last year, the Academic Department of Mental Handicap – jointly with the University of Bristol and Stoke Park Hospital – was established and Senior Lecturer, Dr. Yvonne V. Wiley and Lecturer, Dr. Graham Carter, were appointed.

From 5th to 7th July 1983, the Annual General Meeting of the Royal College of Psychiatrists is taking place in Bristol, home town of the Vice-President of the College. Psychiatry – Bristol Fashion – will be presented by a number of papers by local psychiatrists in sessions held in Pucklechurch, Stoke Park, the Burden Neurological Institute, Glenside and Barrow Hospitals and the Students Union. Very strong national and international contingents of psychiatrists are also participating in the scientific part of the Annual Meeting.

At the Annual General Meeting of the College, Sir Alec Merrison, Vice-Chancellor, University of Bristol, will be elected Honorary Fellow of the Royal College of Psychiatrists.

Dr. Max Valentine, Consultant Psychiatrist from Glenside Hospital, is retiring this month. He will be sadly missed from Bristol's scene. His contribution to psychiatry was immense; he was visiting professor of psychiatry in Iran and worked in the USA. He published a book and a number of papers on psychiatry. We wish him well in his retirement and success with his hobbies and collections.

J.J.

**'Caterpillars into butterflies?'**

What is the natural embryology of a medical paper? My more earnest paper-writing friends tell me that I am out of date. They seem kind and considerate, are often younger, and perhaps they are correct. It is not the topics, the analysis or even the layout of my papers which they find quaint. Or so they say. It is the way they are written that is at fault.

Round the corner there is a magic machine. It is seen in the business adverts and often in the posh Sundays too. A Word Processor. My, I am impressed. So that is how 'personalised' letters come through the letter box extolling the virtues of this car and that encyclopaedia. Well, maybe the Word Processor really is the modern way of producing a paper. The trick is evidently to get the word-processing machine to do the work of rewriting each successive draft. A word here, a transposition there, redo the references and captions to suit the house style and, bingo, it is done. Sounds great, but is this how everybody goes about it?

I read recently that Rudolf Virchow in his years of maturity used to dash his papers off: no corrections, just done first time.[1] No wonder he topped the amazing score of two thousand publications. I bet his referees did not dare to say no. To me his technique sounds more like the obstetrics than the embryology of a medical paper. How do ordinary mortals achieve their papers? Obviously the word-processor enthusiasts see the events as a sort of mammalian embryology: gradual organogenesis here, delete a vestigial paragaraph there, calcify a view here, and effect a limited hyperplasia in that section. The embryo progressively grows fairly predictably, as in the standard embryological texts.

Is all of paper writing like that? My papers, I freely confess, start on all sorts of bits and backs of paper, bills, file covers, reprint margins, computer printouts and even on the insides of match boxes. All these odds and ends fuse, get rewritten several times, and tidied away and hidden by the cats – or so the entire family assures me, become lost, are resurrected and done once again. Eventually they are apologetically slipped in a haze of paper clips and Scotch Tape onto our Secretary's desk. The whole process, to that point, seems much like the mysterious things that caterpillars do before they emerge as butterflies, with bodies neatly packaged into Title, Introduction, Results and Discussion and wings made of Reference and Acknowledgements.

Maybe the Word Processors are right. Does no one else go in for the excitement of metamorphosis? Tell me do, I feel lonely in the cocoon. There is not even any silk munching going on in our bush, although admittedly I cannot hear much in the greenery above the clatter of our departmental Word Processor down the corridor busily printing out the next draft of a colleague's paper. Perhaps it is just a new butterfly's last manicure?

1. Ackerknecht E. H. (1953) *Rudolf Virchow: Doctor, Statesman, Anthropologist.* Madison, University of Wisconsin Press, p. 35.

J. D. DAVIES

